# Book Reviews

**Published:** 1832-05

**Authors:** 


### CRITICAL ANALYSES.

Quæ laudanda forent, et quæ culpanda, vicissim,
Illa prius, cretâ; mox hæc, carbone, *notamus.*—PERSIUS.

*Report on the Chemical Pathology of the Malignant Cholera: containing Analyses of the Blood, Dejections, &c. of Patients labouring under that Disease in Newcastle and London, &c. &c. &c.* By W. B. O'SHAUGHNESSY, M.D. &c. (*Published by Authority of the Central Hoard of Health.*)—8vo. pp. 72. Highley, London.

IT has so frequently happened that those who have sought to explain the nature of disease by the aid of the science of chemistry, have bewildered themselves and their readers by fanciful speculations and hasty and unfounded assumptions, that it cannot be a subject of astonishment if the practical inquirer looks upon all such investigations with much distrust, and but little hopes of improvement. We deem it proper, therefore, to premise our notice of Dr. O'SHAUGHNESSY's very interesting essay, by the assurance that his inquiries have been conducted in the most philosophical and satisfactory manner: he has elicited many most important and novel facts respecting the chemical pathology of the malignant cholera, and in every part, we might almost say in every line of his work, we detect his great anxiety to draw no conclusions but such as clearly and unequivocally arise out of the data he has established.

Dr. O'Shaughnessy divides his report into three principal sections. In the first he gives a concise but careful sketch of the *exact state of our present knowledge* of the chemical composition of the *blood* in the *normal* or *healthy* condition. In the second section, an account is given of all the analytical inquiries yet instituted on the chemical pathology of the malignant cholera; noting the discrepancies between the several experimentalists, and stating the result of his own investigations. In the third division, he inquires into the extent to which these investigations entitle us to form pathological or therapeutical conclusions; and he "endeavours to point out and explain the indications of treatment which they apparently afford."

Dr. O'Shaughnessy commences his remarks on the normal or standard condition of the chemical composition of healthy blood, by observing that the reputed ingredients of blood drawn from the venous system in a state of health may be conveniently arranged and considered in three leading groups: first, those invariably present, in a proportion very little varying from a certain standard, and universally recognised by all chemists and pathologists; secondly, those usually present, but occasionally absent, and perpetually liable to alteration in their quantity; thirdly, those substances, the presence or absence of which in standard blood is asserted by some authorities, and denied, or not recognised, by others.

"Under the first head may be included water, albumen, fibrine, colouring matter, extractive matter, and various saline substances; viz. the carbonate of soda, muriates, sulphates and phosphates of soda and potassa, carbonates of lime or magnesia: phosphates of these earths, and minute quantities of iron, in an unknown state of combination.

"Under the second head may be specified a fatty compound, consisting of an oily and crystallizable matter; also urea, or the peculiar animal principle of urine.

"The third embraces a considerable number of substances: namely, the free acetic acid, carbonic acid, cholesterine, free carbon, and traces of manganese, silica, and copper. (P. 4.)

Attempts have recently been made to demonstrate the uses of the saline ingredients of the blood, and the merit of this investigation, Dr. O'S. says, is almost exclusively attributable to Dr. Stevens, of Santa Cruz, whose clinical statements and experiments have excited the attention of all the scientific physiologists and practitioners of Europe and America.

"Dr. Stevens' experiments may be thus briefly described: He found that dark blood extracted from a vein could not be made to assume the scarlet tint, by exposure to or admixture with the air, except saline matter was present, but that the addition of the minutest possible quantity of a salt, even destitute of oxygen, (such as the chloride of sodium,) immediately restored the red colour. Proceeding on this indication, Dr. Stevens used a combination of saline remedies in his treatment of the secondary period of yellow fever; a disease in which, in this stage, blackness of blood is a most prominent symptom. The results of this practice are described to have been of the most gratifying character." (P. 6.)

The experiments of Dr. Stevens have been characterised by Dr. Prout as apparently unfolding "the germs of immense benefit to mankind;" and Dr. P. has gone a step further than Dr. Stevens, in assigning the particular use of one of the individual salts, namely, the muriate of soda, in the digestive process, during which he thinks it probable that this salt undergoes decomposition; muriatic acid being secreted into the intestinal canal, the free soda remaining in the serum of the blood, which thus becomes endowed with alkaline properties. From the various facts he adduces, Dr. O'Shaughnessy considers it highly probable,

"1. That the saline matters exercise an important, though certainly not defined or ascertained, control over the changes which takes place in blood, during its passage from the venous to the arterial system.

"2. That their absence or diminished proportion is connected in some unknown manner with the production of various diseased conditions.

"3. That the colouring matter of the blood is not the sole ingredient affected by the respiratory process.

"The second group in the broad classification I made of the constituent parts of the blood, embraced the fatty matter and urea. The usual existence of the former in healthy blood is now a universally acknowledged fact. The latter occurs more frequently in diseased conditions, but may still be regarded as an occasional but rare ingredient of the healthy fluid.

"The third and last class of substances asserted to exist in normal blood, is composed of those, the presence of which is either disputed altogether or supported on too limited testimony. I include in this division cholesterine, ozmazome, cruorine, free carbon, acetic acid, carbonic acid, silica, manganese, and copper." (P. 7.)

Dr. O'S. next enters into a critical examination of the opinions of M. Hermann, of Moscow, and Dr. Clanny; and we think he is justified in placing but little confidence in the results these gentlemen have drawn from their analyses of the blood. The analysis of the blood recently published by Dr. Clanny, in which he not only maintains the presence of free carbon, but positively states the quantity to have been 32° in a healthy specimen, and 60° in the blood of a cholera patient, is particularly adverted to by Dr. O'S., who places but little confidence in the statement; which, we may add, is at variance with the results obtained by the most accurate chemists. The only correct point, it appears, in Professor Hermann's analysis of the blood of cholera patients, is the obvious one that there is a deficiency of water. Dr. O'S. then proceeds to the important subject of the comparative quantities of the constituent parts of the blood contained in a given portion of that fluid. To this inquiry he solicits particular attention, as it will be seen in his analysis of blood drawn from individuals labouring under cholera, that an absence or deficiency of some of the ingredients, and a remarkable deviation from the normal proportion in others, constitute, as far as his observations extend, the most remarkable features in the chemical phenomena of the disease, "thus fully verifying the prediction of M. Lecanu: Il peut fort bien arriver que le sang dans les cas pathologiques differe plutôt par un changement dans la proportion relative de ses principes que par la présence de quelque principe accidentel." From the analysis of this distinguished chemist, Dr. O'S. takes his standard for the comparison between the healthy and morbid conditions. This preference he accords to M. Lecanu, because his processes are free from fallacy, and incomparably the best ever devised for the quantitative and qualitative analysis of the blood.

"*Standard Analysis of the Blood, by M. L. R. Lecanu.* (*Journal de Pharmacie*, No. ix., September 1831.

| | | |
|---|---|---|
| Water | 780.145 | 785.590 |
| Fibrine | 2.100 | 3.565 |
| Albumen | 65.090 | 69.415 |
| Fatty matter: | | |
| A. Crystalline | 2.430 | 4.300 |
| B. Oily | 1.310 | 2.270 |
| Colouring matter | 133.000 | 119.626 |
| Extractive soluble in alcohol water | 1.790 | 1.920 |
| Albuminate of soda | 1.265 | 2.010 |
| Muriate of soda; Muriate of potassa; Carbonate, Phosphate, Sulphate } Alkaline | 8.370 | 7.304 |
| Carbonate of lime; Carbonate of magnesia; Phosphate of lime; Phosphate of magnesia; Phosphate of iron | 2.100 | 1.414 |
| Loss | 2.400 | 2.586 |
| Total | 1000.000 | 1000.000 |

In the second section of his work, Dr. O'S. enters upon the *Chemical Pathology of the Malignant Cholera*, and passes in review the analyses performed by Dr. TURNBULL, CHRISTIE, Professor HERMANN, M. FOY, and Dr. CLANNY. His own results are also stated, and to these only shall we direct the attention of our readers.

"These results were based on the examination, first, of three excellent specimens of blood drawn in the malignant cholera; secondly, of one drawn in a case of ordinary, though violent, feculent and bilious diarrhœa; and, thirdly, of the dejected matters of eminently characteristic appearance, (a portion of which is already before the Board,) passed by one of the patients who died of malignant cholera, and whose blood was also examined. After fruitless attempts to obtain *materiel* for analysis in Sunderland, I repaired to Newcastle-upon-Tyne, where the disinterested kindness and scientific zeal of the medical gentlemen of that town at once supplied me with abundant specimens. The first opportunity occurred in the case, an outline of which I subjoin, and which was witnessed by Dr. Macwhirter, of Newcastle; Messrs. Thackrah, Nailor, and Breary, of Leeds; Mr. Nesham, surgeon, of Newcastle; several other gentlemen, and myself.

"*Case and Analysis.*

"*Malignant Cholera.* Mrs. Barras, æt. thirty-nine, widow, of excellent habits, good general health, in rather comfortable circumstances, and residing in a lane adjoining the river, Sandgate, Newcastle, was seized with cramps, epigastric pain, and giddiness, at about ten P.M., on the night of the 17th December. According to the statement of her female friends, she soon after became deadly cold, her countenance altered to the expression of death, she lost all voluntary power, and her eyes became deeply sunk in their orbits. In this state she is reported to have spent the night, having vomited and been purged about six times. A more precise history could not be obtained.

"At nine A.M., on the 18th, she was seen by Mr. Nesham, by whose direction a vein was opened in the arm. The blood issued difficultly, was at first viscid and very dark, but it subsequently assumed a more lively colour. The blood was placed aside, in a small basin, and at eleven A.M. (when I arrived) had separated into a loose, bulky crassamentum, and transparent but unusually viscid serum. The crassamentum having been disturbed and broken up by some of the gentlemen present, the serum only was removed for analysis. The patient passed no urine from the commencement of the attack, until its fatal termination on the night of the 18th December. For the details of the mode in which this specimen of serum was analysed, I beg leave to refer the Board to the Appendix: I shall here only insert the results, giving at the same time, in one column of the table, Lecanu's analysis of healthy serum as a standard for comparison.

"*Specific Gravities.*

"Cholera serum from Mrs. Barras, 1.041. Healthy Serum (Lecanu), 1.028.

"*Comparative Analysis of Standard and Cholera Serum.*

| Ingredients. | Standard of Lecanu. | Serum from Mrs. Barras. | Remarks. |
|---|---|---|---|
| Water | 906.00 | 854.00 | |
| Albumen | 78.00 | 133.00 | |
| Urea | 0. 0 | 0.40 | |
| Organic matter: | | | * Number in 2d column embraces both the organic matter and albumonate of soda. |
| Soluble in alcohol and water | 1.69 | *4.80 | |
| Albumen combined with soda | 2.10 | | |
| Fatty matter: | | | † 2d column includes both the oily and crystalline principles. |
| A. Crystalline | 1.20 | †1.40 | |
| B. Oily | 1. 0 | | |
| Muriate of soda; Muriate of potassa | 6. 0 | 4.00 | |
| Subcarbonate of potassa; Phosphate of potassa; Sulphate of potassa | 2.10 | ‡0.00 | ‡ The 0 in 2d column refers to the carbonate of soda alone. |
| Carbonate of lime | | 0.00 | |
| Carbonate of magnesia; Phosphate of lime; Phosphate of magnesia; Phosphate of iron | 0.91 | §1.60 | § The 2d column here embraces Phosphates of soda, Sulphates of soda, Phosphates of lime, Phosphates of magnesia and iron 1.60 |
| Loss | 1.00 | 0.80 | |
| Total | 1000.00 | 1000.00 | |

"The tabular view thus afforded of the results in this analysis, is interesting in several points of consideration. It shews, in the first place, the absence of a large proportion of water; secondly, the corresponding preponderance of albumen; thirdly, the presence of urea; fourth, the absence of the alkaline carbonate; fifth, a great deficiency of saline materials. I should also add, that the experiments which gave origin to this table were witnessed by Dr. White, of Newcastle; Mr. Hawthorn, the nephew of Dr. Macwhirter, was present when the urea was obtained; and Dr. Macwhirter himself, on entering the room a few minutes after, having been requested to smell the watchglass on which the nitrate of urea was deposited, without any previous knowledge of its nature, compared the odour to that of a '*pot de chambre.*'" (P. 28.)

The second case which afforded Dr. O'S. materials for continuing the inquiry, occurred in the practice of Mr. HAWTHORN: it was one of very severe bilious and feculent diarrhœa, with vomiting. He gladly embraced the opportunity of examining this case, as he was exceedingly anxious to ascertain whether the loss of water in the blood was an invariable sequel of profuse intestinal evacuations. The result shewed the singular fact, that the fluid parts of the blood were, on the other hand, *increased* in this instance. "The consideration of this phenomenon, and of some other peculiarities of the dejections in the malignant cholera, to which I shall advert in the sequel, will probably furnish, at no distant period, a diagnostic test of chemical accuracy, by which the indigenous cholera of Great Britain, to which we have ever been accustomed, can be distinguished from the disease of the same name with which the present winter has for the first time made us too familiar." In this case also the albumen was below the average proportion; the salts were normal in quantity, and the serum preserved its alkaline character.

The next case Dr. O'S. considers to be, in several respects, the most important of the entire: it occurred in the Cholera Hospital, Sandgate, Newcastle, on the 21st December; and was witnessed by Drs. GIBSON, WHITE, MORRIES, several other medical gentlemen, and the author himself.

"*Intensely Malignant Cholera.* The patient, James Dewar, aged thirty-nine, a sailor, of good habits and colossal frame, was attacked, at six A.M., on board the smack Nimble, of Leith, with spasms, cramps, purging, and vomiting of the peculiar fluid, which I need not describe. At nine A.M. he was brought to the Cholera Hospital. Soon after his arrival, he passed a copious characteristic dejection, which was preserved for analysis: he was then given a little ammonia by the house surgeon, Mr. Glenton. Another evacuation followed in about ten minutes, and was also set apart.

"When I saw this patient at eleven A.M., he was perfectly pulseless and cold, his face contracted, and of a tarnished silvery or fishy aspect: he suffered horribly from cramps, and uttered cries like one shouting through a barrel. It was, on the whole, the worst case but one that I witnessed during my stay in the infected districts.

"A little after eleven A.M., some blood was taken from an orifice in both arms, and about eight ounces, dark in colour and viscid in consistence, were with some difficulty obtained; the patient writhing about his bed so constantly that the blood could not be preserved from contact with the atmosphere. This blood was also set aside for analysis.

"Before leaving the ward, and in the presence of Drs. Gibson, White, Morries, and others, I tested the dejections with yellow turmeric paper, and that passed before the ammonia was given changed the colour of the paper to a deep permanent brown. I should add, it had been ascertained that he had taken no medicines previous to his admission into the hospital.

"Notwithstanding the most assiduous attention and active treatment, Dewar died the same day at four P.M.

"The blood was allowed to rest for three hours, and in the interval I proceeded with the analysis of the dejected matters. Dr. White, a gentleman highly skilled in natural philosophy, and to whom I feel much indebted for the patient attention which he bestowed on my investigations, was present at almost the entire of the experiments instituted in the case I now describe.

"The serum and coagulum, when carefully separated and weighed, were in the proportion of 43 serum and 57 crassamentum, by which an extraordinary loss in the aqueous portion of the blood was pointed out. The crassamentum was then examined in the manner described in the Appendix, and found normal in the proportion of its ingredients, so that the addition of a certain quantity of water would have restored it to its original density, proportions, &c. For this reason I have not included the crassamentum in the tabular statement of the analysis.

"The serum was of the sp. gr. of 1.45, and was devoid of the least action on litmus or turmeric papers. I need not dwell on the other steps in the examination, as they did not differ in the least degree from the proceeding adopted in the case of Mrs. Barras, fully detailed in the Appendix No. 1. The whole analysis in Dewar's case was completed the day after his death. The general results can scarcely fail to prove interesting, especially when collated with all the preceding tables, as in the adjoining form.

"*Comparative Analysis of Serum in Health, Malignant Cholera, and Bilious Diarrhœa.*

| Ingredients. | Healthy Standard of Lefanu. | Malignant Cholera. Mrs. Barras | Bilious Diarrhœa. Mr. Hawthorn's. | Malignant Cholera. Dewar. | Remarks. |
|---|---|---|---|---|---|
| | 0. | 1. | 2. | 3. | |
| Water ...... ................ | 906.00 | 854.00 | 921.75 | 866.80 | |
| Albumen ...................... | 78.00 | 133.00 | 61.85 | 124.00 | |
| Urea ........................ | 0.00 | 0.40 | 0.00 | 0.00 | |
| Organic Matter, soluble in Alcohol and Water | 1.69 | *4.80 | * 5.20 | * 4.00 | * Embrace the organic matter and albumen of soda. |
| Albumen combined with Soda..................... | 2.10 | | | | |
| Fatty matter: | | | | | |
| Crystalline ............... | 1.20 | 1.40 | 1.90 | 1.23 | |
| Oily ........................ | 1.0 | | | | |
| Muriate of Soda ......... Muriate of Potassa ...... | 6.00 | 4.00 | 5.00 | 2.17 | |
| Carbonate of Soda ...... | ............ | 0.00 | | | |
| Phosphate of Soda ...... Sulphate of Soda......... | 2.10 | | 2.30 | 0.5 | |
| Carbonate of Lime...... Carbonate of Magnesia Phosphate of Lime...... Phosphate of Magnesia Phosphate of Iron ...... | 0.91 | 1.60 | 1.10 | 0.70 | |
| Loss ...................... | 1.00 | 0.60 | 0.90 | 1.5 | |
| Total .................. | 1000.00 | 1000.00 | 1000.00 | 1000.00 | |

In Dewar's case, the dejections were also analysed. That passed before the ammonia was given was strongly alkaline, of sp. gr. 1007. It was found to be composed of water, mucus, carbonate of soda, and the acetate, muriate, phosphate, and sulphate of the same base: but it contained neither albumen, traces of caseum, or the principles of bile. The solid flaky matter was composed of a mixture of albumen and fibrine.

On the presence or absence of carbonic acid in the cholera blood, Dr. O'S. made no experiment, as he cannot attribute to this agent any of the almost magic properties with which it is endowed by some ingenious speculators.

"One, for example, has gone the length of comparing its functions to 'the action of the spiral spring on the balance-wheel of a watch.' I, on the other, regard the presence of carbonic acid as a sign of the perfect accomplishment of our respiratory and circulatory functions; its absence as an effect low down in the scale of causation, proceeding from derangement of these essential actions. Indeed, instead of adopting the metaphor just quoted, I would rather select for my illustration another part of the timepiece, and compare the carbonic acid to the hands, by whose motions is estimated the regularity or aberration of the internal machinery.

"In short, wherever protracted asphyxia occurs, carbonic acid must be deficient in venous blood, no matter what theory of sanguification we embrace. That such a state of protracted asphyxia forms a primary feature in the *cold blue* cholera, every competent observer must admit, and all the symptoms of the disease unanimously proclaim.

"The summary of my experiments may therefore be described as denoting a great but variable deficiency of water in the blood in four malignant cholera cases; a total absence of carbonate of soda in two; its occurrence in an almost infinitesimally small proportion in one; and a remarkable diminution of the other saline ingredients. Again, in the dejections passed by one of the patients whose blood was analysed, we find preponderance of alkali, and we recover the other saline matters deficient in the blood. Lastly, the microscopic structure of the blood, and its capability of aëration, are shewn to be preserved." (P. 40.)

The third section of the work embraces a brief inquiry into the extent of the pathological and therapeutic conclusions deducible from the preceding statements; and in this most important part of his investigation, Dr. O'Shaughnessy proceeds with the most laudable caution. He admits that the universality of the facts regarding the deficiency of water and absence of the saline matter in the blood, is *not proved*; but he maintains that it is rendered more than *probable* by the circumstance of three experimentalists having arrived at nearly the same results on one of these points, viz. the deficiency of water, by very different modes of investigation. Neither does he regard the alkaline state of the dejections as *proved* to be universal; but it is rendered very *probable* by the fact that Dr. Morries, of Edinburgh, has informed him that Dr. Abbs, of Sunderland, had, in numerous cases, tested the dejected matters with turmeric paper, and always found them possessed of an alkaline reaction. M. Foy, also, has made the same observation at Warsaw, during the irruption of cholera in that city. Having thus shewn that the universality of these facts is *probable*, Dr. O'S. inquires whether, according to the strict rules of induction, we are to consider them as causes or effects? "If as the latter, are they the first effects of the original cause, or should they be placed lower down in the scale; or do they themselves become the proximate causes of any remarkable events in the disease? He regards the alteration of the blood as the *result* of an external impression, but admits that we still remain in darkness as to the mode in which that impression is communicated, although of its nature we have more knowledge to a certain extent: "every circumstance in the semeiology and anatomical pathology of the malady, the kind of its symptoms, the sequence in which they occur, and period of incubation between the application of the primary cause and the appearance of morbid symptoms, all coincide in denoting that an organic poison is the first mover of the consecutive actions."

In Dr. O'S.'s opinion, many facts apparently indicate that the absence of water and saline matters from the blood are not *essentially* connected with the progress or the event of the malady. In some cases, for example, death takes place very rapidly, and without the occurrence of alvine evacuations.

"The most obvious manner in which the loss of water could occasion death, is by the physical obstruction it would necessarily occasion in the passage of the blood through the capillary vessels, especially in the pulmonary circulation. The motion of the blood would, therefore, be confined to the great vessels, which would become distended to excess; gradual asphyxia should supervene, and death be occasioned with all the phenomena of impeded respiration and circulation. How accurately this description coincides with the events in cholera, it is unnecessary for me to point out. In short, this mode of death in this disease might at once be admitted, had we previously accurate ideas regarding the precise density of fluid which the capillary system will receive and permit the passage of, and had we found that the density of cholera blood exceeded this standard. I regret that my inquiries among some distinguished anatomical friends have failed to procure me any conclusive evidence on this subject.

"I shall therefore content myself by expressing my conviction that this deficiency of water in the blood, is, at any rate, in many cases a powerful adjuvant in the production of the fatal event. This view of the case, as well as the general semeiology of cholera, are strongly illustrated by the following brief quotation from the chapter on Hyperœmia, in Andral's splendid work on Pathological Anatomy.

"'When the mechanical hyperœmia is carried to a certain extent, other phenomena may arise as its consequence. Thus the serous portion of the blood may escape from the over-distended vessels, just as water or any other liquid transudes through the permeable sides of a vessel in which it suffers compression. * * * And although these effusions have really nothing active in their nature, yet they are considerably diminished, and sometimes altogether removed by bloodletting, &c.'"

"Giving thus to probable mechanical hyperœmia as much importance as I think it is entitled to receive in the consideration of this disease, I also cannot, or will not, conceal my conviction that the remote cause of cholera may, and frequently does, produce death, independently of this auxiliary, by the unknown agency it exerts on the nervous system." (P. 49.)

The next point to which Dr. O'S. adverts is the probable influence of the absence, or diminution of quantity, of saline matter, in the progress of the malady. From all he has been able to learn on the subject, he infers that the diminution in quantity of saline matter in the blood is not immediately incompatible with life, and that the injury resulting is rather of a chronic character: that it takes hours or days for its production: he, consequently, would not attribute to the absence of the salts any important share in the inducement of sudden death in cholera, but he is inclined to believe such sudden diminution or absence to be closely connected with the fever stage of the malady; and this opinion is at the same time suggested and substantiated by Dr. STEVENS's experiments on the state of the blood in the yellow fever of Santa Cruz.

*Therapeutic conclusions.*

"The commentary I have adjoined to the chemical inquiries, has shown sufficiently that, even though their results were shown to be universal facts, still that their remote causes yet remain open for investigation. I shall not consequently attempt to draw any practical indication from premises which, though perhaps correct, are nevertheless liable to share the fate of all hypothetical speculations.

"The consideration, however, of the presumed effects of these causes, recognized in the alteration of the blood, leads to two important therapeutic conclusions, in the event of the universality of these effects being proved by subsequent inquiries.

"These indications are,

"1st. To restore the blood to its natural specific gravity.

"2d. To restore its deficient saline* matters.

"The first of these can only be effected by absorption, by imbibition, or by the injection of aqueous fluid into the veins. The same remarks, with sufficiently obvious modifications, apply to the second.

"In the milder cases, or preliminary symptoms, ere yet absorption is impeded, I should expect much benefit from the injection of copious enemata of warm water into the intestines. It should, however, be remembered, that in mild cases the necessity for such dilution is not immediately urgent,† inasmuch as the changes which give rise to the indication are not yet completed. The injections may, however, cut short the progress of the sanguineous alteration, which may otherwise supervene.

"At the same time that this diluent injection is practised, a consideration of the state of the patient in each individual case will direct the competent practitioner as to the choice of the other remedies: such as stimulants, opiates, external warmth, &c. which

* "In order to prevent misconception of my meaning, I must again emphatically repeat, that I do not consider this deficiency essential to cholera, but that it occurs as an accidental effect in a vast majority of cases; and that this effect must be obviated before we can accomplish a cure."

† "In the preliminary symptoms, strictly so called, astringents may prevent the inspissation of the blood by the alvine discharges. The best astringent I ever knew the effects of is the following, which saved many lives during the dreadful dysentery which prevailed in Edinburgh in 1829, while I was Dr. Alison's clinical assistant. I have strong expectations that it would be also found of decided utility in the cholera diarrhœa: Take of Opium, twelve grains; Acetate of Lead, twenty-four grains; Conserve of Roses, a sufficient quantity. Make into a mass, and divide into twelve pills, one to be taken as occasion may require."

may be calculated to re-excite the circulation and promote the required absorption; a function so intimately connected with the state of circulation, that the mobility or inaction of one is almost essentially connected with those properties in the other.*

"The tepid water enemata may contain a certain proportion of the neutral salts. It will not be forgotten, however, that in the majority of cases these salts already pre-exist in the intestinal canal.

"In the severe cases in which absorption is totally suspended, and when stimulants, however varied or energetic, fail to re-excite the circulation, I would not hesitate to inject some ounces of warm water into the veins. I would also, without apprehension, dissolve in that water the mild innocuous salts which nature herself is accustomed to combine with the human blood, and which in cholera are deficient. Let it be remembered, that if this experiment be not practised, death is inevitably close at hand, and that the proposal does not rest on idle or frivolous opinions. It should also be remembered that this mode of medication has, in many a desperate disease, been practised with success, and that by some of the most cautious and experienced physicians in the world.

"I beg, however, that I may not be misunderstood, so as to be thought to recommend this proceeding indiscriminately. On the other hand, I would deem that practitioner little better than a homicide who would perform the operation without the sanction of a numerous consultation.

"With respect to the treatment of the fever stage, I would expect much benefit from the frequently repeated use of the neutral salts by the mouth or by enemata, and dissolved in large quantities of tepid water. I should prefer the subjoined combination,† as it imitates to a certain extent the composition of the materials in which the blood is presumed to be deficient. Besides meeting the chemical indication, these remedies will also assist the cure by their aperient properties, &c.

"While this practice is pursued, I would also obey every local indication, and use cold applications, leeches, &c. according to the symptoms of the case." (P. 52.)

In conclusion, Dr. O'Shaughnessy points out the urgent necessity of making further investigations respecting the chemical pathology of cholera. He ingenuously confesses that he has but given the clue to the complete pursuit of the inquiry, and he believes that "its extensive repetition would not only lead to an

* "Since this Report was drawn up, tepid-water enemata have been employed in Newcastle, with the best effects. (See Dr. Gibson's Report, and Mr. Caton's Letters, in the Cholera Gazette, No. 2.) In the cholera of 1688, Sydenham exclusively employed diluents, in order 'to dilute the spirituous parts of the blood;' and, he states, with the utmost success."

† "Take of Phosphate of Soda, ten grains; Muriate of Soda, ten grains; Carbonate of Soda, five grains; Sulphate of Soda, ten grains: dissolve in six ounces of water. The mixture to be repeated every second hour."

increased knowledge of the malady, but perhaps unravel many a mysterious problem in the functions of life and aberrations of disease."

In order to contribute to the acquisition of the desired knowledge, he gives, in an Appendix, some plain and intelligible manipulatory instructions, by which, in the remotest village, any intelligent person may perform such an analysis of the blood and dejected matters as will suffice to confirm or refute the statements and opinions he has submitted in his " Report."

At the end of the Report, mention is made of the analyses, by MM. Rose and Wittelock, of Berlin, of the blood and alvine evacuations in the disease recently prevailing in that city. The results correspond with those derived by Dr. O'Shaughnessy, and thus *the identity of the chemical pathology of the cholera of Berlin and Newcastle is fully proved.* Dr. O'S. has since completed the analyses of four specimens of blood drawn from persons labouring under this disease in its severest forms, and of eighteen examples of the peculiar dejections, obtained from patients in different quarters of London. "The results correspond so perfectly in every particular with those of the Newcastle and Berlin analyses, that to describe them would be but the repetition of the same terms." Dr. Turner, of the University of London, has also made an analysis of a specimen of blood and one of the peculiar evacuations; and his results are quite in accordance with those of MM. Rose, Wittflock, and Dr. O'Shaughnessy. Hence, then, it appears that the cholera of Berlin, Newcastle, and London, is identically the same disease.

In every respect this work claims our warmest approbation: it opens to the consideration of medical practitioners many new views respecting the pathology and treatment of cholera, and we cannot doubt but that our knowledge of the general laws of diseased action will be much improved by a diligent pursuit of the investigation the author has so ably conducted. The besetting sin of the majority of experimental inquirers Dr. O'Shaughnessy has most carefully avoided: he draws no hasty conclusions from imperfect premises; he never raises mere probabilities into positive facts; and he scrutinizes with the same rigid impartiality the result of his own experiments, as he does those of other inquirers in the same field: he thus completely gains the confidence of his reader, as it is evident that his most earnest wish is not to pass beyond the bounds of fair induction. In this point of view, indeed, his "Report" is a model which all experimentalists will do well to imitate, and most especially those who, like him, essay to unfold the laws of disease by the assistance of chemistry. It is scarcely necessary we should add, after the character we have given of it, that this " Report of the Chemical Pathology of the Malignant Cholera" claims the attention of all medical practitioners.

*The Cyclopædia of Practical Medicine.* Edited by John Forbes, m.d. f.r.s., Physician to the Chichester Infirmary, &c.; Alex. Tweedie, m.d., Physician to the London Fever Hospital, &c.; John Conolly, m.d., late Professor of Medicine in the London University. Parts II. III. and IV.—Sherwood and Co. London. (Published in Monthly Parts.)

Although it is obviously impossible that we can enter into a critical examination of each article contained in such a work as the "Cyclopædia," we still feel it incumbent upon us to give to our readers some further account of a publication of such extent and importance. The first part we have already noticed, and, after having looked carefully through the three subsequent parts, we see no reason to modify the favorable opinion we have before given, unless, indeed, it is to express our increased approbation of the manner in which the work is executed. To expect that each article should possess an equal degree of merit, would be unreasonable; and it is clearly impossible that each should be equally interesting to every reader, inasmuch as some subjects must be treated upon in such a publication which can claim the especial attention of but few students or practitioners. When we take up Johnson's Dictionary, or any other lexicon, we do not complain because we find the definition of many words, which all fully comprehend, and concerning which no further instruction is wanting; and it would be equally hypercritical to urge it as a fault, that the "Cyclopædia" includes many articles which have no particular novelty to recommend them.

In the parts before us there are many excellent articles upon subjects which must be interesting to all medical practitioners, and we shall select two or three of them for our present analysis; and first of *Asphyxia*, which is from the pen of Dr. Roget.

The term Asphyxia is restricted to express those cases of cessation of the heart's action which arise from a particular cause, namely, the interruption of respiration; or, to speak more correctly, the interruption of the effect produced by that function on the blood. It is only during a certain short period that this state of suspended animation admits of recovery, on the employment of proper means; but if its duration exceed this period, it is irretrievably fatal. After briefly mentioning the various ways in which asphyxia may be induced, Dr. Roget thus describes the general phenomena of that condition.

"The phenomena consequent upon a deficiency of oxygen in the gas which is in contact with the blood in the lungs succeed one another with different degrees of rapidity, according to the greater or less extent of this deficiency, or according as the obstruction to respiration is more or less complete. The effects of a total interruption to breathing are so quickly fatal as scarcely to allow time for accurately observing the order of their succession. This can best be done when asphyxia is gradually induced.

"The first perceptible effect of impeded respiration is a sensation of distress referred to the region of the lungs, accompanied by a strong desire of fresh air, and by an involuntary effort to dilate the chest by throwing into action, not only the intercostal muscles and the diaphragm, but also those muscles which act as auxiliaries in the same office. This sensation rapidly becomes more and more urgent and painful; it rises to one of extreme agony, which agony, however, is of but short duration, being quickly lost in an overpowering torpor, which steals upon the senses, and bereaves the sufferer of every faculty of consciousness. The struggle is, notwithstanding, maintained for a short time longer; being taken up, in the absence of consciousness and volition, by the natural powers inherent in the system, which still survive. It is in this stage of the progress of asphyxia that various irregular and convulsive movements are excited, both in the trunk and limbs, as if the system were instinctively prompted to these efforts in order to burst the spell about to be cast round it. During these commotions, the veins of the head are observed to swell; the face, and particularly the lips, become blue and livid; the eyes are suffused, and seem ready to start from their sockets. At length these involuntary and fruitless agitations subside into quiescence; the powers appear to be exhausted; nature yields to necessity; and a fatal immobility pervades the system. So complete is this general relaxation of the fibres, that even the sphincter muscles, which always retain their irritability to the last moment, at length give way. Yet the heart continues for a time to propel, though feebly, and with quick vibrations, the venous blood it receives from the pulmonary vessels: but, in a few moments longer, a stop is put to its motion, and all circulation is arrested. The asphyxia is now complete; and the critical moment is arrived when life is fast ebbing at its source, and not an instant is to be lost in resorting to the most energetic means for its recall.

"It is difficult to assign the precise period at which resuscitation becomes impossible; much will depend upon the mode in which the asyphxia has been produced, upon the age and constitution of the individual, and other circumstances hereafter to be noticed.

"Such, then, is the usual course of the phenomena of asphyxia when the abstraction of air has been sudden and complete. When more gradually induced, the sufferings are more protracted; the painful sense of anxiety is accompanied by various feelings referred to the head, such as vertigo, singing in the ears, and scintillations in the field of vision. The extinction of irritability is more gradual, and is not attended with epileptic convulsions. There is also less suffusion of the skin in the case, but a more extensive discoloration over that of other parts of the body, in which red or livid patches often arise.

"On inspecting the body after death from simple asphyxia, we find that it presents externally the marks denoting imperfect oxidation of the blood in the capillary vessels of the skin, which accordingly is dark, and, in some places, purple. These livid spots are not unlike those so commonly met with in bodies that have remained for some time in one position after death from other causes. They may in general, however, be easily distinguished from the different situations which they occupy in the body: for the latter description of patches occur in the most depending parts of the skin, and seem to be the effect of the gravitation of the blood in the vessels. This is not the case with the livid spots arising from asphyxia, which appear to have their seat chiefly in the mucus membrane of the skin; for the corion itself is not much affected. A section of the skin in these parts exhibits numerous points where the blood has been congested in the vessels at least, if not extravasated. The joints are generally rigid in a greater degree than in other cases of sudden death. The features of the countenance have an expression of pain; the eyes are distended and prominent, and the pupils dilated.

"In the interior of the body, the most striking appearance is the great accumulation of blood that has taken place in the pulmonary system of vessels, in the right auricle and ventricle of the heart, and in the great veins which terminate in these cavities; while, on the other hand, the left auricle and ventricle are comparatively empty. The coronary veins of the heart are rendered remarkably conspicuous by their turgescence; and this is also the state of the principal branches of the vena cava. The liver, spleen, and kidneys, are gorged with blood, which may be forced out in large drops by slight compression of the parenchymatous substance of these organs. The blood itself is thick and dark coloured, and is but rarely found coagulated.

"The lungs are in a distended state; and, if not restrained by the adhesions they may previously have contracted with the sides of the chest, they often expand so as to meet, and even overlap one another over the pericardium, when the anterior mediastinum is cut through. Their colour is a dark brown; and, like the organs already mentioned, they readily allow, when compressed, of the exudation of the blood they contain. On opening the trachea, we find its mucous membrane deeply injected with blood; and this appearance is still more strongly marked as we trace its progress into the lesser ramifications of the bronchia. The surface of the membrane is frequently overspread with a frothy liquid, slightly tinged with blood. The fibrous tissue which unites the cartilaginous rings of the trachea and bronchia is also injected with blood, and thereby presents a striking contrast to the white colour of the cartilages themselves.

"When the previous struggle has been violent, indications are afforded of fulness in the vessels of the head: the sinuses and veins of the brain are distended with blood; and a section of the cerebral substance exhibits an unusual number of red points; an appearance which is often accompanied by the effusion of serum in the ventricles. But in cases where death has been attended with but little disturbance, none of these appearances are met with, and all the vessels of the brain are in their natural state.

"The root of the tongue, however, almost always appears as if it had been injected; and its papillæ at this part are remarkably distended. The mucous membrane of the epiglottis and larynx partakes of the affection of that which lines the trachea, and which has been already described." (P. 168.)

*Theory of Asphyxia.* Before the chemical effects of respiration on the blood had been discovered, and when the action of the pulmonary organs in promoting the circulation was believed to be altogether mechanical, the cessation of the motion of the heart in hanging or drowning was ascribed to some mechanical impediment to the transmission of blood through the lungs, arresting it in its course, and preventing its access to the left auricle: but, in Dr. Roget's opinion, the fact may now be considered as fully established, that the real obstacle arises out of the interruptions to those chemical changes which atmospheric air produces in the blood, while circulating in the pulmonary vessels, and which convert it from venous to arterial blood. The blood, which in asphyxia thus retains its venous character, does, in fact, for a time pass through the pulmonary circulation, and is conveyed into the left ventricle, which propels it through the arterial system. But this blood, which is thus substituted for arterial blood, has deleterious properties: it acts, in fact, as a poison on the organs to which it is sent, and by its presence they are deprived of the power of performing their respective functions; sensibility, irritability, together with all the physical and vital actions depending upon these powers, are suspended; and their suspension, even for a minute, is fraught with the most imminent danger to life. Bichat has shewn that the primary effect of the circulation of venous blood is on the brain, and that this effect extends, through the intervention of the brain, to the whole nervous system. "The succession of the phenomena of asphyxia plainly demonstrates that loss of sensibility takes place as soon as this venous blood has reached the brain, and exists for some time before the action of the heart is suspended. This effect on the brain is manifestly the source of the convulsions that ensue, and that indicate the strong impression made upon the nervous system. The patient, as it has been strongly but quaintly expressed, dies *poisoned by his own blood.*" The cessation of the action of the heart was accounted for by Bichat, on the supposition that it was itself paralysed by the deleterious qualities of the venous blood, which, by entering the coronary arteries, penetrated its muscular substance, and destroyed its irritability. To this doctrine Dr. Roget objects: he observes, that, if it were true, resuscitation from asphyxia would be impossible; but since daily experience shews us that the heart may be made to renew its contractions, even some time after they have ceased, we are forced to conclude that that organ still retains, under these circumstances, a considerable share of irritability, ready to be called into action when a proper stimulus is applied.

"In confirmation of the views here given, we may cite the experiments of Dr. Kay,* and also those of Dr. Edwards,† which tend to prove that when venous blood is made to circulate through the substance of muscles, it contributes to support their irritability in a certain degree, although less effectually than arterial blood. Admitting, then, that the heart retains a certain portion of irritability, sufficient to account for the renewal of its contractions under certain circumstances, it would appear that the causes of the cessation of its action may be resolved partly into a deficiency of supply of blood to the left ventricle, consequent upon the paralysis of the capillaries of the lungs, which deprives them of the power of propelling it forwards; partly into its own diminished irritability, arising from the direct effect of the entrance of venous blood into the coronary arteries; and partly also into the participation of the heart with the general deleterious impression made on the whole nervous system, by the circulation of venous blood in the brain and other parts of the system. So intimately are all the vital functions connected together, that it is extremely difficult to assign to each of these causes the real share which it respectively has in the production of the whole of the observed effect. There can be little doubt that any one of them would of itself be sufficient to arrest the movements of the whole machine; and that the derangement of any one of the functions which are here implicated, would necessarily throw all the rest into disorder, and destroy the equilibrium of the system. The first, in point of time, in the series of phenomena consequent upon the suspension of the arterializing process, is the affection of the brain. Were this the sole effect directly produced by the want of oxygen, or superabundance of carbon in the blood, then might asphyxia be ranged under the head of apoplexy; and the subsequent failure of the circulation would be a consequence of the impaired energy of the nervous powers which maintain the energy of the heart. But this can scarcely be admitted to be the sole cause of death; because the motion of the heart in asphyxia is arrested much sooner than it ever is in simple apoplexy. We find, indeed, that in the latter disease the heart continues to beat for many hours, or even days, after the destruction of the faculties of sensation and of consciousness; and it appears at length to stop principally in consequence of the cessation of breathing, which always takes place when the abolition of the powers of voluntary motion has proceeded a certain length. So that, in fact, it may more properly be said that apoplexy proves fatal by inducing a state of asphyxia, than that asphyxia is merely a species of apoplexy, as it has been erroneously classed in some systems of nosology.

* See Edinburgh Med. and Surg. Journal, vol. xxix. pp. 42 and 46.

† De l'Influence des Agens Physiques sur la Vie. Part i. ch. i., and part iv. ch. iv.

"The chief seat of the stagnation of the blood in asphyxia is the minute pulmonary vessels; and this may be inferred from the fact that the whole of that part of the vascular system, and apparatus connected with it, which precedes it in the order of circulation, is distended with blood. Such, we have seen, is the condition in which the pulmonary arteries, the right ventricle and auricle of the heart, the venæ cavæ, and all their branches, are found after death. On the other hand, those portions of the vascular apparatus which follow this point in the order of circulation, are nearly empty of blood; namely, the left auricle and ventricle, the aorta, and all its branches and ramifications. The inquiry into the condition of the pulmonary capillary vessels, which this stagnation of the blood in them implies, would open a wide field of discussion; for it would involve the disputed question of the nature of the action of the capillaries, the power they possess to propel their contents, and the extent in which they contribute to carry on the circulation. Without entering into this discussion, for which this is not the proper place, it will be sufficient to state that, by the expression of paralysis of the capillaries of the lungs, we have merely meant to denote that condition which renders them incapable of transmitting onwards the blood which is sent to them. It might certainly be expected that, on the interruption to the chemical changes which the blood usually undergoes in the lungs, these vessels would be the first to suffer from any noxious property which the blood might thereby acquire; since they are more intimately in contact with it, and more extensively exposed to its action. While we admit, however, that this affection of the pulmonary capillaries is one of the principal causes of the cessation of the heart's action, it is at the same time very probable that the diminution of its energy, occasioned by the circulation of venous blood through its substance, contributes in a great degree to the same effect. It is of great importance, in a practical point of view, to obtain a true theory of asphyxia; since the success of the measures we may adopt for the purpose of restoring suspended animation will depend on its correctness." (P. 170.)

We must pass over the description of particular kinds of asphyxia, that we may be enabled to give the substance of the remarks Dr. Roget offers upon the *general treatment*.

Although the suspension of all the vital actions of the system which takes place in asphyxia has originated from the temporary interruption in a single function, yet the derangement which has followed is of so complicated a nature that the mere reestablishment of the function primarily disturbed is not immediately followed by the restoration of the rest, and by the removal of all the mischief that has been created. The mere introduction of fresh air into the lungs cannot at once restore the action of the heart, and still less that of the diaphragm and the other muscles which are concerned in respiration, because these muscles have lost either the whole or the greater part of their irritability. In the first place, the lungs should be artificially inflated, with as little delay as possible. Next, to excite the powers of the nerves and muscles, stimulants, as warmth, friction, and electricity, are to be applied to various parts of the body: the particular mode of their application depends upon the peculiar circumstances of the case, and is pointed out in the directions for treating the different kinds of asphyxia. Valuable directions for recovering persons under asphyxia have been published by the Royal Humane Society, and of these an abstract is given, together with such additions as have been suggested and recommended by various authorities. The practitioner should bear in mind that "there still exists a period of danger after the breathing has been restored and the circulation reestablished, at which death may take place when we are least prepared to expect it. When animation returns, therefore, the patient should not be left alone, but watched, lest he should require further assistance; for many have been lost for want of attention, who might otherwise have been easily saved." Dr. Roget quotes in illustration the following case, mentioned by Dr. Paris.*

A corporal of the Guards, of the name of Schofield, was seized with cramp as he was bathing in the Thames, and remained for several minutes under water. By judicious assistance, however, he was recovered, and appeared to those about him to be free from any danger, when he was attacked with convulsions, and expired. "Had the respiration been artificially supported at this period, so as to have maintained the action of the heart until the black blood had returned from the brain, it is probable that the life of the soldier might have been preserved."

Delirium sometimes follows the return of sensation; and in every case, even after the functions have been restored, we must be on our guard against the effects of morbid reaction which gives rise to various inflammatory and febrile states.

Asthma. This article, by Dr. Forbes, claims particular notice on account of its practical excellence. In popular language, every chronic shortness of breath is termed asthma, and some medical writers, up to a recent period, have sanctioned the extensive and ill-defined application of the term. Dr. Forbes confines himself chiefly to that variety of disordered respiration called *spasmodic asthma*, and which may be briefly defined a difficulty of breathing recurring in paroxysms, after intervals of comparative good health, and usually unaccompanied by fever. The "history" of asthma, as here given, is very instructive, and we regret that we cannot do more than refer to the leading points which Dr. Forbes discusses.

In the treatment of asthmatic patients, the practitioner should carefully investigate their general state of health; for although, in the intervals between the paroxysms, apparent good health may be enjoyed, "their state will rarely, if ever, stand the test of the

* Life of Sir Humphrey Davy, 4to. ed., p. 69.

inquiry of the physician." Some permanent local disease of the organs of respiration or of some other organ, or some disturbance of function, may generally be detected on examination.

"In examining the pathology of asthma, we have to consider not merely the phenomena of the paroxysm, but of the interval also; and to investigate the condition not merely of the respiratory organs, but of all the other organs. In this inquiry we shall find that, although there may exist in every case one or more conditions which more especially characterize the disease, and without whose presence asthma could not be said to exist, yet that there are many other conditions of the system, or of particular parts, which only occasionally accompany the more specific phenomena, and yet constitute, in the cases wherein they do occur, the most important features of the disease. It is upon these accidental or contingent phenomena that the means calculated to prevent, relieve, or cure, asthma, more particularly depend; the knowledge of them, therefore, is most essential to the practitioner; and every attempt at exposing the pathology of the disease must be extremely imperfect which does not take them into account. With these reservations, which are only such as practical physicians find necessary, in almost all cases, when applying the results of theory to the actual phenomena of disease, we are disposed to coincide in the opinion of Willis, Hoffmann, Cullen, and others, respecting the proximate cause of the asthmatic paroxysm. The theory of these great men, as is well known, was, that the asthmatic fit consists essentially in a spasm of the muscular fibres of the bronchi. In admitting the existence of this spasm, however, as essential to constitute the disease, we are very far from looking upon it, in a practical point of view, as in every case the most important part of the affection. On the contrary, as we have just stated, many of the other morbid conditions, general or local, which commonly precede or co-exist with this phenomenon, are of infinitely greater importance, both pathologically and in practice; but, so long as these conditions are unaccompanied by this peculiar affection of the muscular fibres of the bronchi, the disease is not termed asthma.

"Although strong doubts have been entertained by many eminent pathologists, not merely as to whether such a spasmodic affection of the bronchi actually took place in asthma, but even as to whether it was possible for it to take place in any case, we have no hesitation whatever in maintaining the affirmative of the proposition as true, both in the general and particular instance. Not merely the preponderance of authority, but anatomical investigation, pathological phenomena, analogy, and probability, are all in its favor.

"The older authors, particularly Willis and Malpighi, demonstrated to their own satisfaction the muscular fibres in the larger bronchi. But the facts mentioned by them have been not merely confirmed but greatly extended by the minuter researches of modern anatomists." (P. 186.)

Various eminent authorities are referred to in proof of the muscular structure of the bronchi, a fact which has been sometimes disputed. As far as our own observation extends, we are led to coincide entirely with Dr. Forbes, that "the main phenomena of the asthmatic paroxysm are precisely such as would result from a morbid contraction of the bronchial muscles, and several of them seem quite inexplicable on any other principle." Some pathologists have maintained that the paroxysm depends upon a spasmodic affection of the muscles of the glottis in particular, and others have referred it especially to spasm of the *external* muscles of respiration. Dr. Forbes adduces many powerful, and we believe convincing arguments, to shew that the main site of the spasms which constitute the asthmatic paroxysm, is the bronchi; but he does not maintain the opinion that the spasms are exclusively confined to these parts. He thinks it certain that not only all the parts already mentioned, but others still more remote, are frequently involved in the same disordered action. He observes, that in all spasmodic diseases there is a disposition towards extension of the spasm from the original or principal site, and that this fact is perhaps more easily explained on physiological principles than the restriction of the spasm to one part or one set of muscles could be." In all these cases, if the nervous centres are not primarily affected, they invariably become so subsequently; and the circumscription of the local manifestation of the spasm to the muscles first disordered thence becomes very improbable." We know also that muscles whose actions are at all associated are very liable to suffer generally when one or more of the class are morbidly affected; and Dr. F. admits that the muscles both of the larynx and the chest are frequently involved in the progress of the paroxysm: they are, however, he believes, in almost every case affected secondarily, and he therefore concludes it to be impossible to subscribe to the opinions of those who wish to make them the chief site of the asthmatic paroxysm. In a few instances, the paroxysm may depend upon muscular spasm of parts otherwise healthy, "but it is not to be doubted that, in the great majority of cases, the spasm not merely affects parts previously diseased, but that the phenomena of the paroxysm are partly dependent on, and greatly modified by, these very lesions coexisting with the spasm, aggravating it, and in turn being aggravated by it."

Dr. Forbes divides asthma into two classes, "according as there exists a sound or a diseased state of the bronchial membrane in the intervals of the paroxysms." Cases of the first class he terms *nervous asthma*, and those of the second *catarrhal asthma*. Persons subject to the first species of the disease are characterized by the extreme susceptibility of their nervous system: in common language, they are nervous. Nervous asthma frequently occurs in hysterical females, and is indeed often only one of the multiform aspects of hysteria. Mere moral causes are frequently sufficient to induce a paroxysm in those who are subject to asthma, and "it seems at least probable that similar causes, when more intense, or when the individual susceptibility is greater, may give rise to the disease in persons not previously subject to it." The records of practical medicine contain innumerable examples of the influence of the nervous system over the asthmatic paroxysm. Dr. PARRY's posthumous works are referred to for some curious cases of this kind: in one, severe dyspnœa, with irregular action of the heart, was instantaneously produced, in the presence of Dr. Parry, by mental agitation. The paroxysm lasted eight or nine days, and terminated with considerable mucous expectoration, shewing the power of a mere nervous affection to produce, by its continuance, local disease. In another case of old and well-marked asthma, the same physician witnessed the instantaneous removal of the paroxysm by fright. Dr. Forbes refers to another singular instance of the influence of the nerves in producing asthma, without any local disease of the lungs. It is a kind of spurious asthma, which attacks opium eaters on being suddenly deprived of their habitual dose; and no doubt for the same reason that delirium tremens attacks the spirit drinkers of colder climates.

"The patient is seized with extreme breathlessness, exactly resembling that of asthma; the countenance being haggard, the pulse rapid, and the eye such as we should expect to find in a patient affected with phrenitis. If the disease is not relieved, it proves fatal in the course of a few hours. Some opium given in time will immediately relieve all the symptoms."* (P. 189.)

For the description of the other species, *catarrhal asthma*, we must refer to the work. The following brief recapitulation includes some of the principal points which seem established.

"1. In the disease properly termed asthma, there is always present a spasmodic contraction of the muscles of the bronchi, and sometimes a similar state of the muscles of the trachea, larynx, and external muscles of respiration.

"2. In a small proportion of cases, the spasmodic stricture may take place (idiopathically or symptomatically) without any previous disease of the affected parts.

"3. In the great majority of cases the spasmodic constriction is dependent on a pre-existing irritation of the mucous membrane of the air-passages.

"4. Phenomena of a very similar character are sometimes the consequence of a congested state of the mucous membrane of the air-passages, without any attendant spasm.

"5. The congested or tumefied state of the mucous membrane almost invariably accompanies the paroxysms, whether this state be a cause or a consequence of the spasm.

"6. The violence of the paroxysms is modified no less by the degree of the congestion than by the degree of the spasm; a great

* HENDERSON, in Edinb. Journal, vol. xxiv. p. 51.

congestion with slight spasm producing, probably, the same result as a slight congestion with a great degree of spasm.

"7. In some cases, the tumefied or congested state of the bronchial membrane passes off entirely with the spasm, without any exhalation from the vessels or augmented secretion from the mucous follicles. More commonly there is a simultaneous relaxation of the spasm of the muscular fibres, and an exhalation from the mucous coat. This exhalation most commonly puts an end to the disease for a time; not unfrequently, however, the congestive passes to a more permanent state of inflammatory irritation, under the form of pulmonary catarrh or bronchitis." (P. 193.)

The morbid anatomy of asthma, and the diagnosis and prognosis, are next considered. As asthma, strictly so called, scarcely ever leads to a fatal result in its early stages, few opportunities are found of investigating the pathological anatomy of it in its simple state. In the greater number of the recorded dissections of asthmatic patients, the morbid appearances are almost always to be looked upon either as the consequence of the disease or as concomitant lesions, not more closely connected with the asthmatic paroxysm than as remote or exciting causes, and constituting no essential parts of the pathological state. Willis, Laennec, Ferrus, Corvisart, Leroux, Lerminier, and many other celebrated pathologists, state that it is frequently impossible to detect, upon the most careful dissection, any organic lesion whatever to which asthma can be attributed.

The *diagnosis* of asthma is seldom difficult to the experienced practitioner; but the student will peruse with advantage the remarks Dr. Forbes offers upon this subject. The *prognosis* in asthma needs no formal discussion. "The disease hardly ever proves fatal as asthma, that is, in the paroxysm; but its frequent recurrence not merely aggravates the pathological states in which it has originated, but leads directly to the production of other diseases." We may observe, however, that the paroxysm of asthma is as frightful to behold as it is painful to bear, and that the young practitioner might easily enter into the alarm of immediate dissolution during its severity, which is so often felt by the attendants, although the patient himself is seldom apprehensive of danger.

Dr. Forbes admits what we would fain, but cannot, deny, that there are few diseases less amenable to the interference of art; and, as far as relates to the cure of asthma, he would still sum up in the words of old John of Gaddesden, "Et primo sciendum est, asthma in senibus non recipere curationem, nec in alia ætate nisi difficulter, maxime si sit antiquum."

We cannot dwell particularly on the enumeration of the predisposing and exciting causes of the disease, as we have but just space to give a slight sketch of the remarks Dr. Forbes makes upon the *treatment of asthma.* What is here said upon this subject applies almost exclusively to the chronic forms of the disease, as

"it will be recollected that the disease termed acute asthma is either a variety of bronchitis or a violent congestion of the pulmonary mucous membrane, and that it is to be treated on principles applicable to such pathological states, with little regard to the spasm which accompanies it."

*Treatment in the Paroxysm.* Bloodletting is neither generally useful nor safe: it may occasionally be necessary as an auxiliary to other means, or as a measure of precaution against the ill consequences likely to be produced by the paroxysm on other parts; but it never, Dr. Forbes believes, puts an end to the paroxysm, and much less does it cure the disease. Cupping is sometimes useful when there is much cerebral congestion. *Narcotics, Antispasmodics,* &c. have been generally prescribed, but with little or no benefit. "In most cases, the only portion of the disease which such remedies are calculated to relieve (the spasm) is conjoined with, and dependent on, a pathological condition of the bronchial membrane, over which they have little or no control." In cases of pure nervous asthma alone, opium, and similar remedies, are safe or prudent. *Stramonium* is highly spoken of as one of the temporary remedies. We remember two cases in which this medicine proved highly useful when all others had failed, in relieving the severity of the asthmatic paroxysm: it is smoked in the manner of tobacco. Dr. Forbes observes, however, that it sometimes fails, and sometimes decidedly aggravates the dyspnœa. *Lobelia Inflata* has lately rivalled stramonium in public estimation, but its pretentions are said to rest on much slighter grounds. *Coffee* has been recommended by Sir John Pringle, Floyer, and Dr. Bree. Dr. Forbes ranks it with other narcotics and stimulants, and, therefore, he places no reliance on it as a general remedy. Ipecacuan, squills, refrigerants, and derivants, are all occasionally useful during the paroxysm of asthma.

In concluding this part of his subject, Dr. Forbes especially directs the attention of the practitioner to the vast importance of paying strict attention to the initiatory or catarrhal stage of asthma. At this period he advises warm pediluvia, warm diluents, and diaphoretics on going to bed, and sometimes purgatives; and particularly the vapour bath. The treatment in the interval between the paroxysms must, of course, vary very much according to the peculiar circumstances of each case. Upon this subject, Dr. Forbes makes many good practical remarks.

In the other parts of the "Cyclopædia" now before us, there are many other papers we should have much pleasure in giving an analysis of, and which would yield much instructive matter for our readers; but for the present we must conclude our notice of the work, with again expressing our conviction that it will become a standard book of reference for medical students and practitioners.

*Thoughts on Cholera Asphyxia.* By ROBERT BREE, M.D., F.R.S, Fellow of the Royal College of Physicians, &c.—8vo. pp. 71. John Wilson, London.

WE readily admit that it is not the common fate of medical works to pass through "five editions," especially "five large editions," or, even at the end of seven and thirty years, to be able to boast of a "fifth edition:" still, with all due allowance for the exultation of successful authorship, we could have excused a threefold iteration of this phenomenon in less than thirty pages. But let it pass: we can venture to predict that the present *brochure* will not be called on to submit to a similar ordeal. Nevertheless, it is worth perusal, and is perhaps above the par of most of those ephemeral productions which the present excited state of the public mind with regard to cholera has forced into unnecessary existence; for, although there is little that we think of importance to the point in question, the pamphlet contains several interesting cases well deserving to be placed on record.

Dr. BREE has long been favorably known to the profession for his researches into the pathology of asthma, and his general inquiries into the causes and phenomena of disordered respiration; but, like many others whose attention has been especially directed one way, when persuaded to look another, he seems to contemplate the novel prospect in the same position, and through the same medium, which, how true soever it may have been for many, for many objects it must needs be false.

*Ramollissement* has latterly become a favorite pathological term: we do not know that it conveys any different idea from what might be equally well expressed by the old technicality *mollities*, or the indigenous word *softening:* but it is neither English nor Latin, and therefore it is adopted.

We know not any structure that may not, under certain circumstances, become disorganized and softened; and, without doubt, these changes have, until lately, been too little regarded: in another part of our present number will be found some interesting cases of softening of the spleen; and to a similar cause, i. e. to ramollissement, or softening of the respiratory nerves, and especially of the ganglia, does Dr. Bree attribute the "state predisposing to the reception of Cholera Asphyxia."

"If we give our consideration to the probable seat of the first reception of the cause of this disease, we cannot deny that the largest surface, and of an organ the most important to the human frame, is most likely to receive the miasmata of the poisoned air in this epidemic. The nervous tissue of the lungs is most susceptible of an attack, and, making a part of the pulmonary system, is most liable to be first affected by the poison. The sympathetic nerve more particularly extends its branches through the strictures of the heart and lungs: and the phrenic nerve, passing through the diaphragm, continues its influence for health or disorder by entering into the abdomen, and connecting itself by anastomosis with the cœliac ganglion. Thus, it may be also said that the continued development of disease enlarges the seat of the cause, and extends the cause itself, as the nerves receive the malignant principle in the secreting tissues of the viscera of the abdomen.

"The scheme of local influence and progress of the nerves that are in question in this inquiry, begins, we may say, with the fifth and sixth pair that make the great intercostal; but the most important must be considered the eighth, or sympathetic nerve; the par vagum, which gives off very soon in the chest, the recurrent nerves and the right and left branches that form the cardiac plexus; the pulmonic and œsophageal plexuses. This nerve next enters the abdomen, where it seems to take the construction of plexus in the most important manner. We have the cœliac plexus, the hepatic, the splenic, and renal, plexuses, in forming which, the great intercostal gives its communication and assistance. The ganglions are, then, a new source of nervous power; and, according to Munro, there is in the composition of these ganglions an exceeding intimate mixture of minute nerves and vessels. The plexuses, combined of nerve with nerve, are also new sources of nervous energy, and are fitted to detach again branches to various parts." (P. 5.)

"The nervous branches, proceeding from the eighth pair; the recurrent nerves, and the cardiac plexus, extend and ramify through the heart and lungs, and all their organic communications: through all of which the influence of this sedative poison is diffused, and transmitted.

"By this calamity, a paralytic state is thus permitted to fall on the viscera of the chest and belly, and partially on the powers of the voluntary muscles. If the loss of sense and motion is not inflicted, the more general effect of spasmodic actions, commonly called cramps, is suffered with torture through the external muscles and limbs of the extreme parts of the body. These painful cramps accompany the morbid actions of the secreting vessels, and the disordered actions of the stomach and bowels; and they seem to be more specially derived from the intestinal canal, and the condition of its coats, than from any other source of nervous sensibility and communication." (P. 9.)

Such being our author's pathology of the predisposing causes of cholera, he proceeds to argue that, as in certain cases of debility of the respiratory system, and especially in cases of asthma, much benefit has been derived from the use of tonics, particularly of preparations of iron, therefore ferruginous remedies will probably be found beneficial, not only as medicines, but as prophylactic means; and not only in asthma, but in cholera likewise.

We do not pretend to affirm that Dr. Bree's speculations may not be correct: indeed, they are more plausible than most of the thousand and one hypotheses which have been raised, many without even the shadow of a foundation; but as we do not find any sections which have shewn the requisite phenomena recorded, nor any cases of cholera, in which the proposed treatment has been tried, reported, we shall defer until a future time our remarks on the cholera department of this pamphlet, and proceed to select for extract two or three pulmonic diseases, the accounts of which, as we have already hinted, are interesting.

"*Case of Convulsive Asthma.* Very recent opportunity, under the eye of the intelligent and learned Dr. Bostock, has given me evidence of the carbonate of iron being both effectual and safe, in the most tender subject of convulsive asthma with congestion, manifested both in the abdominal viscera and pulmonary organs. Miss Isabella Yates, a child of six years old, niece to Dr. Bostock, had suffered these attacks every month or six weeks for five years. In all her attacks, besides those symptoms that are usual in convulsive asthma, she was affected with a sense of great heat and soreness in the throat. She had not taken the carbonate of iron. I have not been surprised, on some occasions, at a reluctance being felt at making this oxide a remedy in delicate subjects in asthma; but in this patient, so tender, and of such sensibility, and under the pressure of the actual fit of convulsive asthma, I found no objection; and I hold that even such a subject may experience the happy removal of spasms and dyspnœa from the use of carbonate of iron, in the most severe fits of convulsive asthma. A paper of six grains of this oxide was given every three hours, in the height of her distress, and when the mother entertained the most fearful apprehension, from the urgent severity of the attack. From the first dose, of six grains, relief was perceptible, and in three more doses the breathing was easy, and the acute attack entirely removed." (P. 26.)

We do not apprehend that any danger can in this instance arise from the indeterminate manner in which the same medicine is called by different names, viz. oxide and carbonate of iron, because the carbonate, under ordinary circumstances, loses its carbonic acid very speedily, and degenerates into the state of oxide; but if, as in some cases it does happen that the carbonate becomes the appropriate remedy, (for this will often remain on the stomach, and be efficacious, when the oxide fails,) then the indiscriminate use of terms signifying two different drugs should certainly be avoided.

"*Nervous Debility of the Lungs. Case.* Mr. T. Gray, being then a member of a numerous family, was twenty years ago well known, and is now living in health, in Mount street, pursuing his business, of a dealer in horses. This young man was greatly affected with cough and expectoration, at times accompanied by painful dyspnœa. This had been his disorder for more than a year, when his reduction of flesh and strength having gone on, to the alarm of his friends, he applied for the opinions of various physicians. Relief was not obtained; and the fact of a rapid consumption, which had been the subject of apprehension to his family and friends, was authorized also by his medical advisers. They indeed had left him with the prospect of an incurable disease of the lungs. When I was applied to for my advice, I had the possible advantage of still hoping, with a new opinion respecting his case. The progress of pulmonary consumption must have been undoubted, if the symptoms I now saw had been accompanied with pyrexia and a febrile pulse from the beginning of the disease. A febrile pulse, at this part of its progress, with inflammatory symptoms of pain, or of tubercles, must have marked the phthisical habit. Guided rather by sanguine hope, than a clear view of the origin and causes of this complaint, I ventured to take the symptoms with favorable expectation. I attributed his pulse, now ranging from ninety to 110, to have become so quick from the actual weakness of the organs of respiration; I considered that this weakness, and laxity of muscular fibre, were connected with a more than proportionate morbid debility of the nerves themselves. His large expectoration appeared to be muco-purulent; his night sweats were caused by the defect in nervous energy. If it were a fortunate conjecture that, in the first part of the progress of this disorder, such nervous debility had been passed over without adequate consideration; and that symptomatic, but vague, appearances were too much relied upon, I might have some hope of advantage from a tonic treatment; and particularly as a spasmodic action was still apparent in the dyspnœa that affected him. In this view, Mr. G. might be said to be suffering a nervous debility of the lungs, without destruction of substance.

"This hope was in fact realized. The means used were entirely of forms of iron, bitters with tincture of iron, the myrrh mixture of Griffith, rhubarb with carbonate of iron. We soon observed considerable amendment from this plan, on which the patient regularly advanced to recovery, and may now be seen in Mount street, in perfect health." (P. 27.)

*Case of Sir Charles Mordaunt*, marking the distinction between the hectic waste of consumption and the chronic decline of nervous powers.

"*Debility of Pulmonary Nerves.* It was in the year 1805 that I was desired to attend a Warwickshire gentleman, who was an object of great interest to his county, and to a large circle of private friends. He had been affected with cough and asthma for a considerable length of time, so that he had carried, for the last two years, all the marks of a consumptive habit. He had profuse expectoration and sweats, but not at regular hectic periods. He suffered pains of the chest and sides; a troublesome tendency to diarrhœa; and the most distressing dyspnœa in the night, which assumed a convulsive form at periods of a week or ten days. He was so emaciated as to make an unusual picture of leanness; and by his friends he was reckoned decidedly in danger of dying in a few months. Relief, even, was scarcely promised him, but, as a probable source of hope, he was advised, by the highest medical authorities, to go to the warmer climate of Portugal. He pursued this measure, and went, in the most debilitated condition, on board his ship. I believe he was first taken to Gibraltar; he was afterwards moved into Spain and Portugal upon a litter, without alleviation of his pulmonary disorder or weakness. After he had staid in these climates without deriving advantage, till he had lost all hope of benefit, he again committed himself to a ship for his return home. I had his assurance, upon his return, that he had kept his bed all the way, and was carried from it when he came to England, with his linen wrappers adhering to his skin, such was the state to which he was reduced.

"Considerable disappointment at his dangerous state of health was at this time felt by the county, from the necessity of supplying a vacancy in the representation; and at this period I was desired to take him under my advice. It is useless to spend description and many words in reporting a case that, to the opinion of all observers, was that of far-gone consumption. I had, however, said that this gentleman was likely to acquire an improved condition of strength. His friends seized upon this step of encouragement, and with ardour would have his name proposed as representative for Warwickshire. I was, to my surprise, often and anxiously asked, by the supporters of his cause, for fresh and fresh assurances of his progress, and they shewed great surprise and satisfaction, when I answered to many of them, that Sir Charles Mordaunt was not in a pulmonary consumption. I had, in fact, conceived expectation of much more progress of his future improvement of health, at the period of this public anxiety, than I had ventured to mention in my first opinion. This honourable and grateful man, in less than two months from his return to England, was himself sensible of increased power of body, without increase of cough, or the other symptoms of equivocal safety. He was therefore now brought into the centre of his rejoicing party in the County Hall, from a distance in Wales where I had recommended him to go, with a course of tonic medicine, and other strengtheners of his stomach, and that he might be exempt from hurry and public curiosity.

"It was now no longer doubtful that he would be able to stand in his place on the hustings, as candidate to represent his county. He was authorized by my assurances, to expect still more powers of body, and the decrease of symptomatic weakness in parts of it. If, indeed, he were not in a consumption, the expenditure of much flesh and corporeal strength was to be compensated by a different course of raising his system, too greatly let down. I could therefore anticipate more and more renovation of bodily health and strength.

"I was present when Sir C. M. appeared in his place, in the County Hall, and expressed, in forcible terms, which I am not capable of intruding upon the reader, the greatful assurance of his recovery to health, and his power of engaging himself to execute the public business of this important county. He omitted no expression of his feeling that could gratify his physician and his anxious friends on this occasion of universal popular rejoicing. Sir Charles Mordaunt was unanimously elected member for Warwickshire in several successive parliaments, and died only a few years since.

"I had never said that he was to be made a perfectly healthy man, but that he would be capable of active public business in parliament; and, above all, that he was not cured of a consumption, because it had not been his disease. I believe he must have sat in parliament eighteen or twenty years, suffering in his last year chiefly from asthma and a local affection.

"In this case of debility of the nerves of the lungs, the agents used at the very first, and always resorted to when means were required of relief, were the various preparations of iron, and such tonic remedies as cure the asthma, and debilities of nervous structure, but which would have been destructive in tubercular consumption or hæmoptoë." (P. 61.)

